# Prediction of Antimicrobial Resistance in People Living With Cystic Fibrosis Using Machine Learning

**DOI:** 10.1002/mco2.70894

**Published:** 2026-08-12

**Authors:** Junrong Jiang, Akhil Naik, Dilip Nazareth, Dennis Wat, Graham Hyde, Jo Fothergill, Sarah Benabidallah, Gregory Y. H. Lip, Sandra Ortega‐Martorell, Ivan Olier, Freddy Frost

**Affiliations:** ^1^ Liverpool Centre for Cardiovascular Sciences at University of Liverpool Liverpool John Moores University and Liverpool Heart & Chest Hospital Liverpool UK; ^2^ Guangdong Provincial Geriatrics Institute, Guangdong Provincial People's Hospital (Guangdong Academy of Medical Sciences) Southern Medical University Guangzhou China; ^3^ Department of Cardiovascular & Metabolic Medicine, Institute of Lifecourse & Medical Sciences University of Liverpool Liverpool UK; ^4^ Artificial Intelligence and Digital Technologies Research Institute Liverpool John Moores University Liverpool UK; ^5^ Adult CF Centre Liverpool Heart & Chest Hospital NHS Foundation Trust Liverpool UK; ^6^ Clinical Infection, Microbiology and Immunology, Institute of Infection, Veterinary and Ecological Sciences University of Liverpool Liverpool UK; ^7^ Danish Center for Health Services Data Science Research, Department of Clinical Medicine, Aalborg Centre Liverpool John Moores University Aalborg, Denmark, Liverpool UK

**Keywords:** antimicrobial drug resistance, cystic fibrosis, electronic health record, machine learning, microbial sensitivity test

## Abstract

Antimicrobial resistance (AMR) is a growing challenge in people living with cystic fibrosis (pwCF), who often experience chronic lung infection and repeated antibiotic exposure. Because routine antibiotic susceptibility testing often takes several days, treatment is frequently started before current resistance profiles are available. We assessed whether routinely collected electronic healthcare records (EHR) could predict antibiotic resistance in sputum cultures from adults with CF. In this retrospective single‐center study, 12,618 sputum cultures from 209 pwCF between 2012 and 2022 were linked with 63,823 days of intravenous antibiotic exposure, spirometry, demographic characteristics, microbiology results, and historical resistance data. Different models were trained and evaluated with patient‐level splitting and cross‐validation to predict resistance to ciprofloxacin, ceftazidime, meropenem, piperacillin/tazobactam, and tobramycin. Extreme gradient boosting showed the most consistent performance with AUCs of 0.75–0.80. Model discrimination was broadly similar in cultures with and without Pseudomonas aeruginosa, except for ceftazidime and meropenem. Shapley Additive Explanations (SHAP) suggested that longer term resistance history was more informative than recent results. These findings support the feasibility of using EHR‐derived data to estimate AMR before culture results are available, but external validation, broader antibiotic exposure data, and assessment of temporal dataset shift are needed before clinical use.

## Graphical abstract

Using routinely collected electronic healthcare record data from patients with cystic fibrosis, machine learning models predicted antimicrobial resistance in sputum cultures before susceptibility results were available. Extreme gradient boosting showed consistent discrimination across five commonly tested antibiotics, with AUCs of 0.75–0.80. Overall, SHAP analysis suggested that longer term resistance history was more informative than recent results, supporting earlier personalized antimicrobial decision‐making.

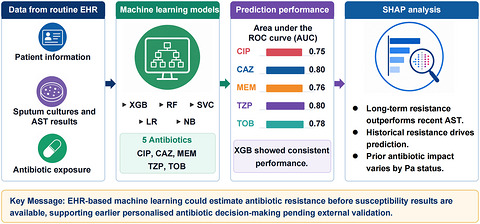

## 1 Introduction

Cystic fibrosis (CF) is an autosomal recessive inherited disease caused by defects in the CF transmembrane conductance regulator (CFTR) gene [1]. CF is a multi‐organ disease, but is classically associated with chronic lung infection, inflammation, structural lung damage, and ultimately terminal respiratory failure [2, 3, 4]. Persistent lung infection by difficult‐to‐treat pathogens such as *P. aeruginosa* is common and often requires a high burden of antibiotic treatment, including long‐term oral prophylaxis, and suppressive inhaled antibiotics [5]. Additionally, people living with CF (pwCF) experience acute exacerbations of their chronic infections requiring prolonged treatment with high‐dose intravenous antibiotics.

The increasing global burden of antimicrobial resistance (AMR) is major concern for pwCF and a recent James Lind Alliance priority setting partnership identified reducing or mitigating AMR as a key priority [6]. In order to mitigate or modulate the development or progression of AMR in pwCF, we must first be able to predict it. While on a population level, AMR is clearly linked to antibiotic use [7], there is limited understanding of how to predict this on an individual basis, particularly in the chronic infection setting. For example, little is known about whether cumulative antibiotic exposure or more recent antibiotic exposure is most relevant to standing AMR and how specific combination antibiotic exposures might modulate the subsequent development of AMR.

Routine antibiotic susceptibility testing (AST) often takes days to read out and as such does not usually inform antibiotic selection at the time of initiation, particularly for acute exacerbations of chronic lung infection. Machine learning (ML) approaches have gained much prominence in risk prediction and diagnosis in a range of medical settings and have been used to support empirical antibiotic therapy decisions in critical care environments [8]. However, to the best of our knowledge, to date there have been no applications of ML to the prediction of AMR in the chronic lung infection setting.

Considering that predicting AMR prior to acute antibiotic administration may allow personalized approaches to antibiotic selection optimized to avoid AMR, we investigated the ability of ML models incorporating routinely collected data from mature electronic healthcare records (EHR) to predict AMR in pwCF. By linking a decade of routine AST results with prior intravenous antibiotic exposure and clinical data, we found that ML models, particularly extreme gradient boosting (XGB), predicted resistance to five commonly used antibiotics with reasonable discrimination. Model performance was broadly consistent across cultures stratified by *P. aeruginosa* status, and explainability analyses suggested that longer term resistance history was more informative than recent AST results. These findings highlight the potential value of EHR‐derived prediction tools as a proof‐of‐concept approach to support earlier, more individualized antibiotic decision‐making in chronic lung infection, while requiring external validation before clinical implementation.

## 2 Results

### 2.1 Baseline Characteristics

**FIGURE 1:**
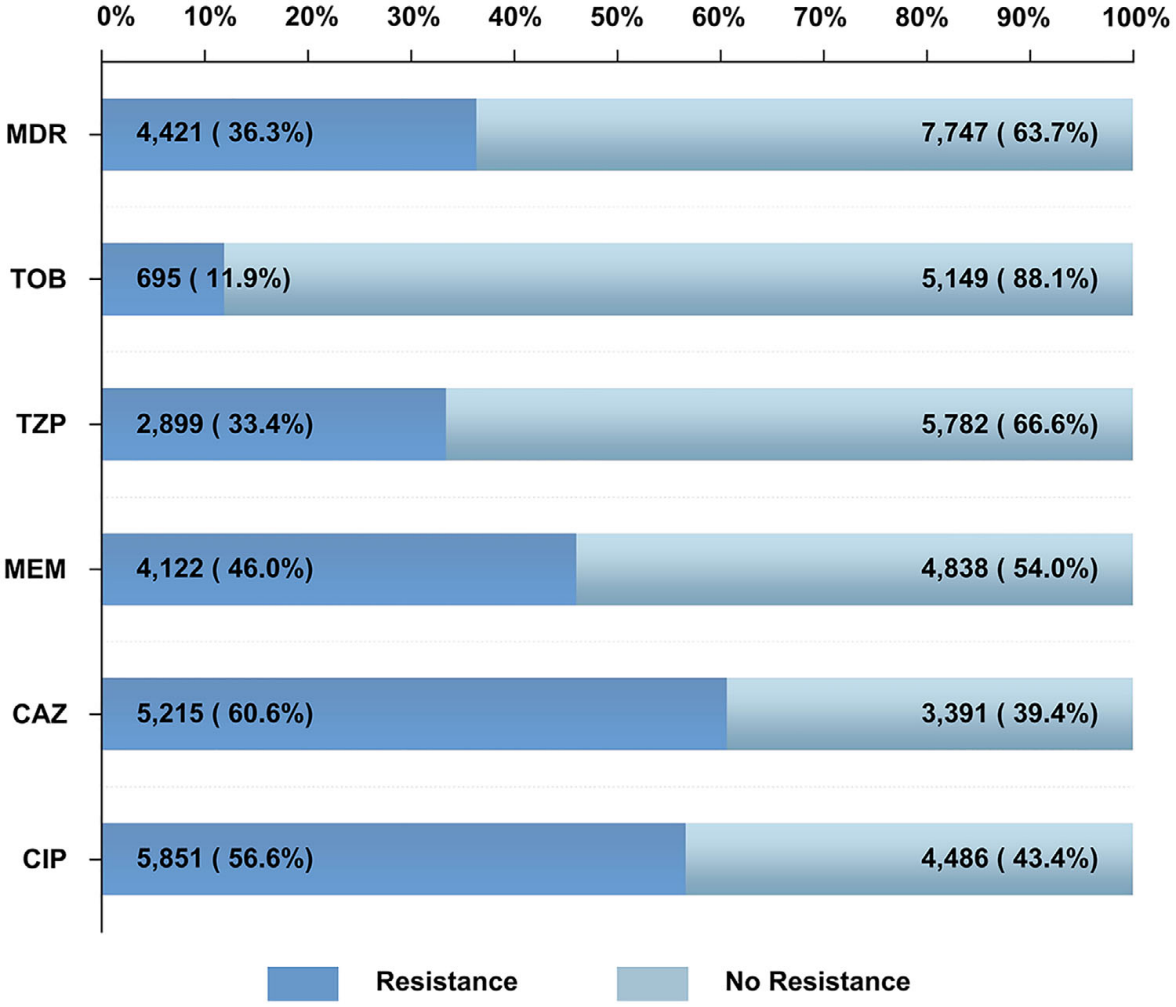
Distribution of antimicrobial resistance and nonresistance among sputum samples. The stacked bar chart shows the proportions and numbers of samples classified as resistant or nonresistant for multidrug resistance and for each of the five commonly tested antibiotics. CAZ, ceftazidime; CIP, ciprofloxacin; MDR, multidrug resistance; MEM, meropenem; TOB, tobramycin; TZP, piperacillin/tazobactam.

To describe the study cohort and the burden of antimicrobial resistance, we first summarized the baseline characteristics of the included pwCF. Among the AST results of 12,618 sputum samples, resistance to ciprofloxacin and ceftazidime were more common, with a resistance rate of 56.6% and 60.6%, respectively, while 46.0% and 33.4% of the samples were resistant to meropenem and piperacillin/tazobactam, respectively. The resistance to tobramycin was the rarest, with a resistance rate of 11.9% (Figure 1).

The baseline characteristics of pwCF are summarized in Table 1. Overall, the median age was 33 years, 42.1% were male, and 55.5% were diagnosed with cystic fibrosis–related diabetes (CFRD). Note that 34.9% carried the Liverpool epidemic strain (LES) of *P. aeruginosa*. The median frequency of samples with valid AST testing per patient was 50, with a median follow‐up time of 6.7 years. The usages of different antibiotics for the 209 pwCF are shown in Table S1. Meropenem, colistimethate, ceftazidime, and tobramycin were the top four most commonly used antibiotics, with median cumulative usage of 38, 42, 24, and 25 days, respectively.

**TABLE 1:** Baseline characteristics and prevalence of resistance during the study period for 209 included people living with cystic fibrosis.

	Total (*n* = 209)
Age (years), median (IQR)	33.0 (28.0–39.0)
Male, *n* (%)	88 (42.1%)
CFRD, *n* (%)	116 (55.5%)
LES, *n* (%)	73 (34.9%)
ppFev1, median (IQR)	53 (40–70)
No. antibiotic susceptibility tests, median (IQR)	50 (26–82)
Follow‐up (years), median (IQR)	6.7 (4.6–8.3)
Prevalence of tobramycin resistance, median (IQR)	2.9 (0.0–14.5)
Prevalence ceftazidime resistance, median (IQR)	55.9 (25.5–79.4)
Prevalence piperacillin/tazobactam resistance, median (IQR)	16.0 (0.0–46.2)
Prevalence meropenem resistance, median (IQR)	39.3 (5.4–61.3)
Prevalence ciprofloxacin resistance, median (IQR)	56.1 (25.8–73.3)

*Note*: Categorical variables are reported as *n* (%), whereas continuous variables are reported as median (IQR) because of the nonparametric distributions.

**Abbreviations**: CFRD, cystic fibrosis–related diabetes; IQR, interquartile range; LES, Liverpool Epidemic Strain; ppFEV1, percent predicted forced expiratory volume in 1 s; pwCF, people with cystic fibrosis.

### 2.2 Machine Learning Models Predict Individual Antibiotic Resistance With Reasonable Performance

**FIGURE 2:**
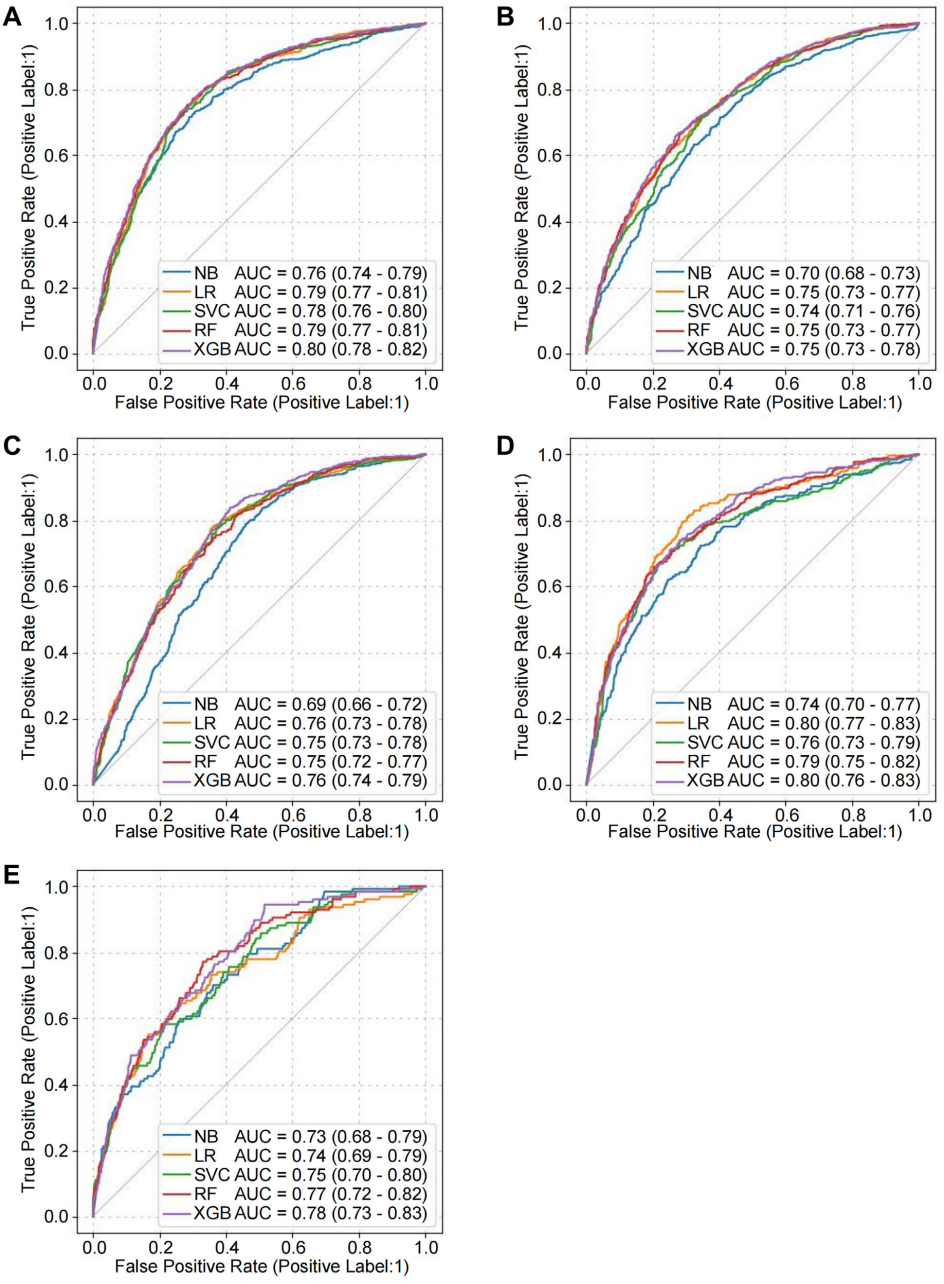
Receiver operating characteristic curves of the machine learning models for predicting antibiotic‐specific resistance. Panels show model performance for resistance to (A) ceftazidime, (B) ciprofloxacin, (C) meropenem, (D) piperacillin/tazobactam, and (E) tobramycin. AUC values are presented with 95% confidence intervals. AUC, area under the receiver operating characteristic curve; LR, logistic regression; NB, naive Bayes; RF, random forest; SVC, support vector classifier; XGB, extreme gradient boosting.

ML models, including naive‐bayes, logistic regression, support vector classifier, random forest, and XGB, were trained to predict resistance to individual antibiotics. After training with fivefold cross‐validation, the ROCs of different models in predicting resistance to each antibiotic were shown in Figure 2. Figure 2A–E presents the model discrimination for ceftazidime, ciprofloxacin, meropenem, piperacillin/tazobactam, and tobramycin, respectively.

In general, XGB seemed to be the most consistent across all outcomes. The full confusion matrixes of predictive performance in resistance to individual antibiotics are presented in Table 2. Briefly, the AUC for predicting resistance to ciprofloxacin was 0.75 (95% CI: 0.73–0.78); for ceftazidime, 0.80 (95% CI: 0.78–0.82); for meropenem, 0.76 (95% CI: 0.74–0.79); for piperacillin/tazobactam, 0.79 (95% CI: 0.76–0.82); and for tobramycin, 0.78 (95% CI: 0.73–0.83). These results indicate reasonable discrimination for predicting resistance to the five commonly tested antibiotics.

**TABLE 2:** The confusion matrix of the best model for predicting individual antibiotic resistance.

AST outcome	Observed	Predicted		Prec	Rec	Acc	F1	AUC	95% CI
		S	R						
CIP	S	651	363	0.68	0.73	0.69	0.70	0.75	0.73–0.78
	R	290	772						
CAZ	S	616	289	0.69	0.79	0.73	0.74	0.80	0.78–0.82
	R	177	650						
MEM	S	679	477	0.50	0.84	0.67	0.63	0.76	0.74–0.79
	R	96	488						
TZP	S	1036	403	0.36	0.73	0.72	0.48	0.80	0.76–0.83
	R	83	228						
TOB	S	499	530	0.18	0.93	0.53	0.30	0.78	0.73–0.83
	R	9	118						

Abbreviations: Acc, accuracy; AST, antimicrobial susceptibility testing; AUC, area under the receiver operating characteristic curve; CAZ, ceftazidime; CI, confidence interval; CIP, ciprofloxacin; F1, F1 score; MEM, meropenem; Prec, precision; R, resistant; Rec, recall; S, susceptible; TOB, tobramycin; TZP, piperacillin/tazobactam.

### 2.3 Differential Model Performance in *P. aeruginosa* and Non‐Pseudomonal Organisms

**FIGURE 3:**
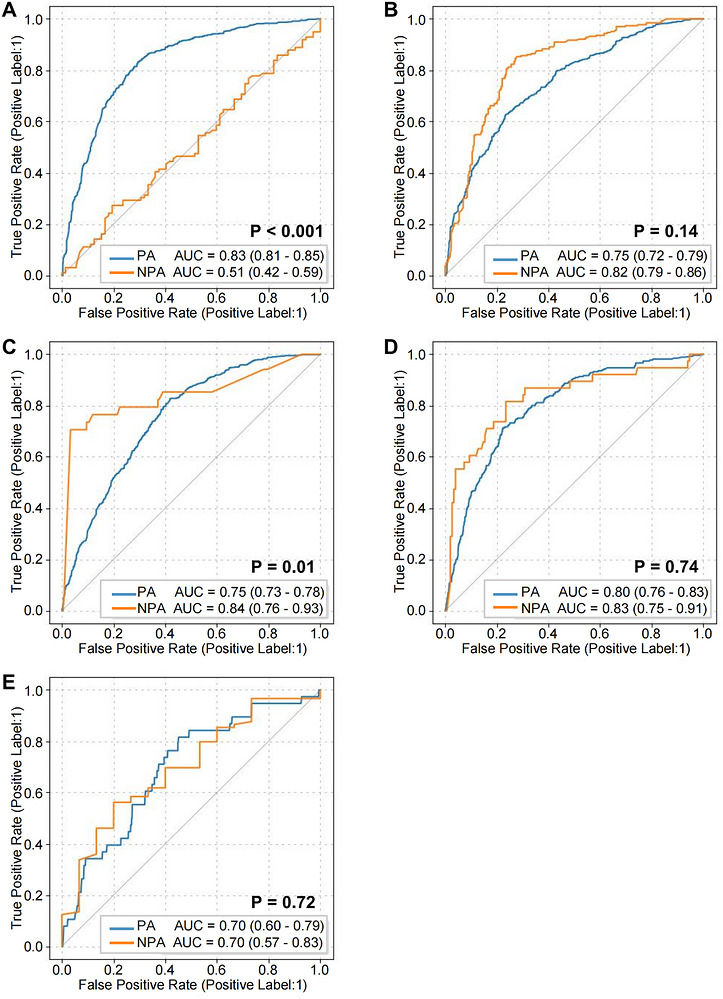
Subgroup‐specific receiver operating characteristic curves stratified by *P. aeruginosa* status. Panels compare model performance between *P. aeruginosa*‐positive and non‐*P. aeruginosa* cultures for predicting resistance to (A) ceftazidime, (B) ciprofloxacin, (C) meropenem, (D) piperacillin/tazobactam, and (E) tobramycin. AUC values are presented with 95% confidence intervals, and *p* values indicate differences in model discrimination between groups. AUC, area under the receiver operating characteristic curve; NPA, non‐*P. aeruginosa*; PA, *P. aeruginosa*‐positive.

To determine whether model performance differed according to the presence of the key CF pathogen *P. aeruginosa*, we stratified by the presence or absence of *P. aeruginosa* and termed the groups “Pa” and “Non‐Pa,” respectively. The confusion matrixes of predictive performance in resistance to individual antibiotics between the two groups are presented in Table S2. Figure 3A–E compares the ROC curves between the Pa and Non‐Pa groups for ceftazidime, ciprofloxacin, meropenem, piperacillin/tazobactam, and tobramycin, respectively. We observed differential predictive performance between groups, where for predicting the resistance to meropenem, the performance of the ML model was better in Non‐Pa Group than Pa Group (*p* = 0.01), while for predicting the resistance to ceftazidime, the performance of the ML model was better in Pa Group than Non‐Pa Group (*p* < 0.001). There was no difference in predicting the resistance to ciprofloxacin, piperacillin/tazobactam, and tobramycin between the two groups. Overall, these subgroup analyses suggest that model performance was broadly comparable by *P. aeruginosa* status, with antibiotic‐specific differences for ceftazidime and meropenem.

### 2.4 Additive Explanatory Approach Reveals Determinants of Antibiotic‐Specific AMR

**FIGURE 4:**
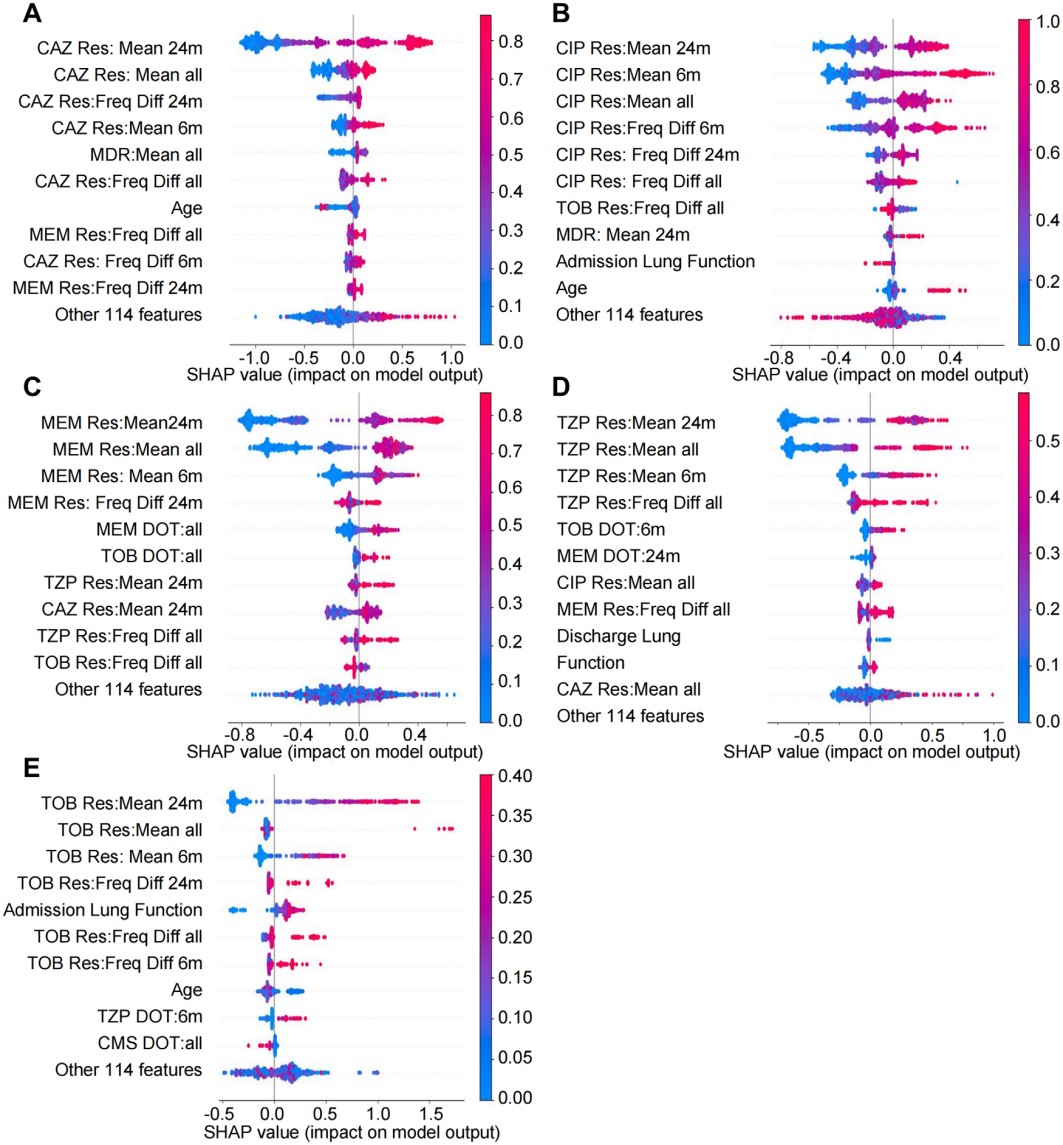
SHAP summary plots showing the most important predictors of antibiotic‐specific resistance in the overall cohort. Panels show feature contributions for predicting resistance to (A) ceftazidime, (B) ciprofloxacin, (C) meropenem, (D) piperacillin/tazobactam, and (E) tobramycin. Each dot represents an individual sputum sample. The x‐axis shows the SHAP value, indicating the direction and magnitude of each feature's effect on the model output; color indicates the feature value. CAZ, ceftazidime; CIP, ciprofloxacin; CMS, colistimethate sodium; DOT, days of therapy; Freq Diff, frequency difference between resistant and susceptible AST results; MDR, multidrug resistance; MEM, meropenem; Res, resistance; SHAP, Shapley Additive Explanations; TOB, tobramycin; TZP, piperacillin/tazobactam; 6m, preceding 6 months; 24m, preceding 24 months; all, the whole available period before the index culture. Other 114 features represent the combined contribution of the remaining features not shown individually.

To explore which variables contributed most to model predictions, we used Shapley Additive Explanations (SHAP) analysis to assess feature importance for antibiotic‐specific resistance outcomes. SHAP summary plots are presented in Figure 4 to visualize the relative contribution of each variable to model performance and allow comprehension of the relative contribution of each variable to the model predictions. Top 10 most relevant variables are presented in descending importance, where variables in each plot seem to be influential. Each horizontal line is made up of dots representing individual sputum samples, and the color of the dot signifies the variable value with respect to that sputum sample. The position on the x‐axis demonstrates the direction and degree of influence on the prediction such that those with SHAP values > 0 make resistance more likely, whereas those < 0 make nonresistance more likely. Interestingly, a consistent pattern was observed whereby longer term antibiotic resistance (total historical resistance patterns or preceding 24 months resistance patterns) was consistently more important than shorter term resistance patterns in predicting all individual antibiotics. This pattern was also seen in the Pa group (Figure S1); conversely for the *Non‐Pa* group (Figure S2), antibiotic usage records and the more recent (6 month) resistance seemed to be more valuable in predicting resistance to individual antibiotics.

This approach also allowed examination of the respective effects of individual agents with regard to collateral resistance. Here, we found that total prior colistimethate use was important for the prediction of ciprofloxacin resistance in both subgroups, albeit with inverse effects. For example, in *the P. aeruginosa* group, historical colistimethate use was associated with increasing ciprofloxacin resistance, whereas in the non‐*P. aeruginosa* groups, increased historical colistimethate use was associated with ciprofloxacin sensitivity. In the Pa groups, total usage of piperacillin/tazobactam was an important feature for tobramycin resistance prediction and vice versa, tobramycin was an important feature for piperacillin/tazobactam resistance. Taken together, the SHAP analyses suggest that historical resistance patterns and selected prior antibiotic exposures were key contributors to model predictions, with potentially different feature patterns between Pa and Non‐Pa cultures.

## 3 Discussion

In this study, ML models can predict AMR in sputum samples from pwCF with reasonable accuracy using routine data extracted from EHR. With the expanding data stored within EHR and the increasing applicability of ML in healthcare, this approach has the potential to inform antibiotic selection for people living with chronic lung infection. These findings are consistent with other prototype models demonstrating that off‐the‐shelf techniques applied to routinely available clinical and laboratory data can yield clinically meaningful predictions to support empiric antimicrobial therapy [9].

Cystic fibrosis is characterized by chronic lung infection and despite the recent availability of highly effective modulator therapy leading to improvements in overall well‐being [10], data from previous successfully modulator treatments suggest up to 60% of people will still be chronically infected with *P. aeruginosa* [11]. As such, cumulative lifetime exposure to antibiotics may therefore increase in some pwCF, and AMR will become a life course issue. Efforts to improve the identification and mitigation of AMR are therefore much needed, and this remains a key priority identified in the refreshed James Lind Alliance process recently undertaken with the CF community [6]. Illustrative of the burden of AMR in this population, we observed multi‐drug resistance in over one‐third of samples in this cohort.

Current practice for pathogen detection involves respiratory sampling at hospital visits and during pulmonary exacerbations, with expectorated sputum culture established as the standard of care [2]. The frequent microbiological surveillance for harmful or transmissible pathogens required as part of routine care for pwCF means that a huge amount of historical culture and AMR data exists for each individual. However, at the time of treatment initiation, the current AST profile is often not available as routine sputum culture and sensitivity can take 48–96 h to yield results. This represents a potential opportunity for the use of ML approaches to predict subsequent AST results, and we explored this using decade‐long AMR data supplemented with linked antibiotic usage records. Although previous studies have demonstrated the predictive value of ML in acute infection [12, 13, 14], predicting AMR of chronic CF lung infection within the same person is more challenging. To our knowledge, this is the first application of such an approach in the chronic lung infection setting, and overall, we found ML methods could predict resistance to antibiotics with good accuracy with AUCs ranging up to 0.75–0.80. This proof‐of‐concept could ultimately have clinical relevance by giving clinicians a prediction of the current resistance profile of an individual patient at the time of antibiotic decision making.

Given *P. aeruginosa* is the major pathogen in adults living with CF, we stratified samples based on its presence or absence and found that there was no difference in predicting resistance to individual antibiotics between the *Pa* group and *Non‐Pa* group except ceftazidime and meropenem. This was perhaps surprising given *P. aeruginosa* is notoriously adaptable in its ability to generate AMR and can undergo extensive pathoadaptations in the CF lung [15, 16, 17]. Co‐infection is common in people with CF and is not accounted for in traditional clinical resistance testing but has been shown to influence resistance patterns as well as pathogenicity of a range of CF pathogens [15, 18, 19]. An explanation for our findings here may therefore be that interactions and cross‐talk between different constituents of the lung microbiota may result in shared or convergent AMR evolution trajectories and this requires further evaluation. Beyond that, resistance predictions interpreted in a pathogen‐specific manner may be more clinically useful, supporting more personalized empiric therapy for *P. aeruginosa* versus non‐*P. aeruginosa* infections.

Pathoadaptation also leads to population diversity, which may not be appreciable in routine clinical AST where often only one isolate is routinely tested. These issues could be explored and refined in future work by including greater number of isolates of *P. aeruginosa* or by including genotypic information about previous *P. aeruginosa* isolates. Indeed, in the acute bloodstream infection setting, ML approaches incorporating whole‐genome sequencing demonstrated excellent accuracy for prediction of phenotypic sensitivity [20]. To the best of our knowledge, this approach is yet to be applied in the chronic infection setting.

In this study, XGB consistently demonstrated strong discrimination across multiple antibiotic‐specific prediction tasks, likely because it can capture nonlinear associations and interactions among prior antibiotic exposure, historical resistance patterns, and patient characteristics without extensive feature engineering [21]. Although gradient boosting is less transparent than linear models, clinical interpretability can be enhanced using SHAP to quantify feature contributions [22]. For the *P. aeruginosa* group, clinical characteristics were rarely important and in general, longer term AMR patterns rather than more recent AMR history tended to be more relevant to subsequent AMR. The converse was true in the non‐*P. aeruginosa* group. The chronic nature of *P. aeruginosa* lung infections in CF and consequent high cumulative antibiotic exposures [11, 23] may explain these findings and suggest that short‐term antibiotic selection strategies may not be relevant to long‐term AMR trajectories. Instead, long‐term antimicrobial stewardship and AMR mitigation strategies are likely needed to be able to reduce the risk of AMR in *P*. aeruginosa infection and its deleterious clinical consequences for pwCF. Individualized antibiotic regimens designed to optimize clinical outcomes while mitigating AMR are one such potential strategy, albeit untested to date.

Interestingly, specific antibiotic usage patterns were associated with collateral resistance. For example, tobramycin cumulative usage was an important variable for meropenem and piperacillin/tazobactam resistance. Colistimethate was a feature of importance for ciprofloxacin resistance but exhibited an opposite effect in *P. aeruginosa* and non‐*P. aeruginosa* groups. Conversely, tobramycin resistance reduced the chance of ciprofloxacin resistance, supporting a recent study which demonstrated that resistance to ciprofloxacin can be associated with collateral sensitivity [24]. Together, these findings highlight the potential for an individualized approach to antibiotic selection where collateral sensitivity and resistance patterns are exploited to mitigate long‐term AMR. These SHAP‐identified relationships are clinically plausible and may reflect co‐selection under repeated antipseudomonal treatment pressure. In *P. aeruginosa*, reduced β‐lactam susceptibility can arise through a combination of mechanisms including AmpC derepression, reduced outer membrane permeability (e.g., OprD loss), and efflux pump upregulation, which together can contribute to cross‐resistance across multiple β‐lactams and carbapenems [25, 26]. In addition, future work in association rule mining techniques may further illuminate co‐resistance and cross‐resistance patterns across antibiotics for understanding collateral resistance trajectories [27, 28].

Beyond antibiotic‐driven selection pressure, broader changes in CF management may influence AMR trajectories. Highly effective CFTR modulator therapy may reduce exacerbations and alter airway microbiology, thereby shifting antibiotic exposure patterns and selection pressure over time [29]. In parallel, updates to AST protocols and interpretive breakpoints may change resistance classification across calendar years and introduce temporal dataset shift, potentially affecting model calibration and performance [30, 31]. Local epidemiology and antimicrobial stewardship practices (e.g., P. aeruginosa prevalence, preferred empiric regimens, and escalation thresholds) may also vary across centers, limiting transportability and necessitating external validation and, where appropriate, recalibration. To strengthen clinical applicability, future work will validate the models in independent, ideally multi‐center cohorts and link EHR data with outpatient prescribing/pharmacy records to incorporate oral and inhaled antibiotic exposure.

This study has several limitations. First, as a retrospective analysis of routinely collected clinical and microbiology data, incomplete exposure capture and residual confounding are possible. Antibiotic exposure was limited to hospital‐recorded intravenous (IV) courses, and oral/inhaled antibiotic use—common in CF care—was not available; future work will link outpatient prescribing/pharmacy records to incorporate these exposures and update the models. Second, all data were derived from a single specialist CF center, so model performance and feature patterns may reflect local epidemiology, microbiology workflows, and prescribing practices; external multi‐center validation (and recalibration where needed) is required before clinical deployment. Third, the decade‐long study period may introduce temporal dataset shift due to evolving CF management and laboratory AST protocols/breakpoints, which could affect calibration over time. Finally, if integrated into clinical workflows, robust data privacy and security safeguards will be essential.

ML models can predict AMR in sputum samples with reasonable accuracy using simple data extracted from EHR. Future work should explore the incorporation of more detailed clinical and pathogen data to improve model performance, particularly for *P. aeruginosa*. With the expanding data stored within EHR and the increasing applicability of ML in healthcare, further developing and refining this approach has the potential to inform antibiotic selection for people living with chronic lung infection.

## 4 Materials and Methods

### 4.1 Study Population and Data Collection

The Adult CF Centre at Liverpool Heart & Chest Hospital NHS Foundation Trust is one of the largest CF centers in the UK and houses a mature comprehensive electronic healthcare record (AllScripts, Veradigm) with over 10 years of data. As part of routine clinical service evaluation, microbiology surveillance, and registry data collection, a dataset including clinical characteristics, admission data, intravenous antibiotic usage, sputum culture results, and AST is automatically maintained.

For the time‐period 2012–2022, a total of 16,800 positive sputum cultures in 290 pwCF with antibiotic usage records for 72,964 intravenous antibiotic days were available. We characterized data at the level of the sputum sample such that for each sputum sample, data available included AST results, linked clinical metadata, and previous antibiotic exposure. Antibiotic exposure was summarized at the level of the antibiotic agent used with prior total cumulative days usage and cumulative use frequencies recorded for the whole study period as well as specifically for the preceding 6 and 24 months time periods. Sputum cultures without contemporaneous linkable antibiotic exposures were excluded. We implemented a prespecified workflow to address bias and data leakage in retrospective EHR data, including handling of missing AST values and patient‐level data splitting for model evaluation. This study was reported in accordance with the TRIPOD (Transparent Reporting of a multivariable prediction model for Individual Prognosis Or Diagnosis)+AI statement.

### 4.2 Definition of AMR

In total, AST data for 33 different antibiotics was available in 13,364 tested samples (see Table S3). Clinical AST testing was performed as per local guidelines and in accordance with contemporaneously relevant UK or EUCAST guidelines [32]. The precise combinations of antibiotics tested depended on local clinical laboratory protocols and we therefore only explored resistance to the five most commonly tested agents, namely, ciprofloxacin, ceftazidime, meropenem, piperacillin/tazobactam, and tobramycin. We dichotomized based on the presence/absence of resistance. According to all results of AST in each patient, the mean resistance to each antibiotic, that is, the proportion of resistance to each antibiotic in all tests, was calculated. Pathogens resistant to three or more antibiotics were considered multi‐drug resistant (MDR).

### 4.3 Handling of Missing AST Results

**FIGURE 5:**
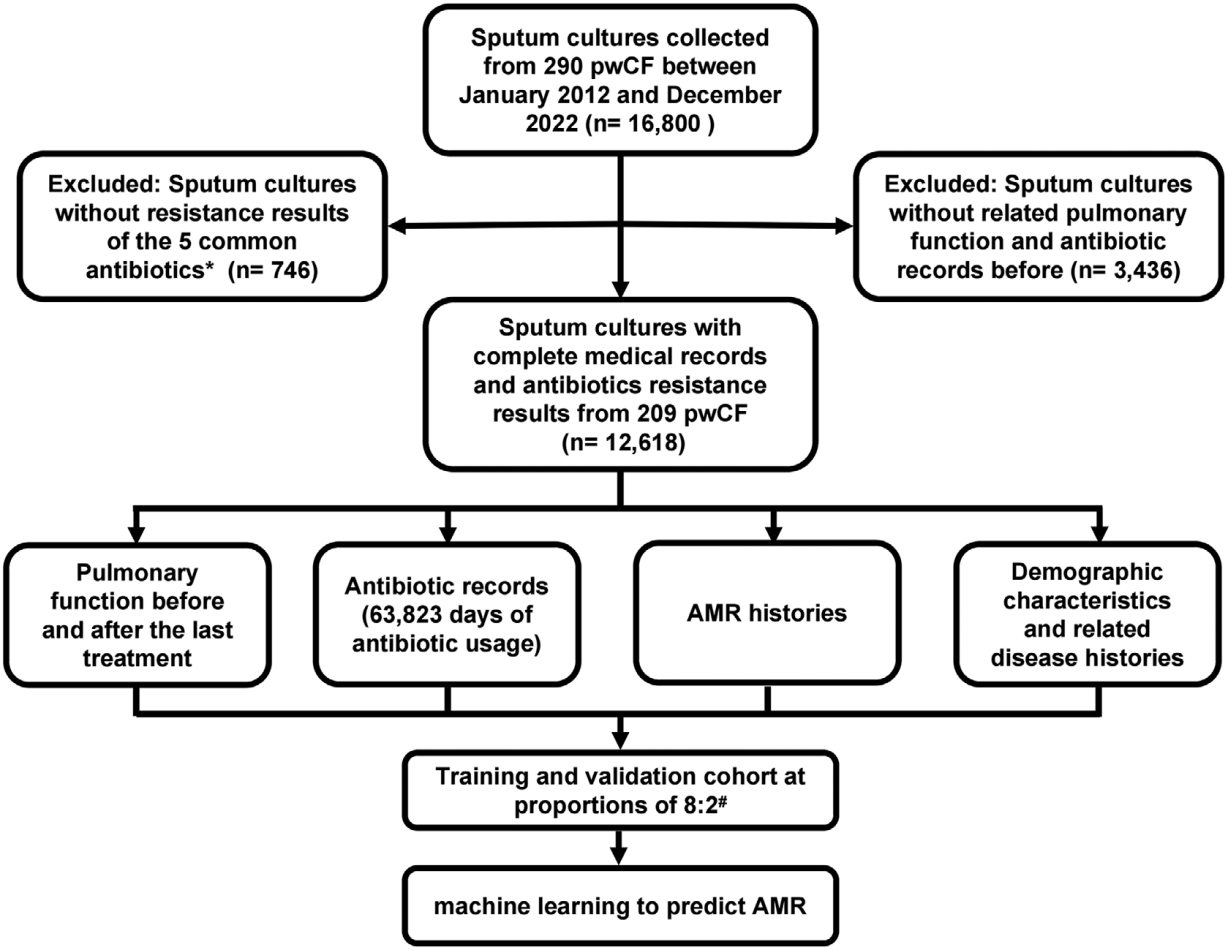
Study flowchart. Sputum cultures collected from pwCF between January 2012 and December 2022 were screened according to the availability of antibiotic resistance results, pulmonary function records, and antibiotic exposure records. The final dataset included sputum cultures with complete medical records and antibiotic resistance results, which were linked with pulmonary function, antibiotic records, AMR history, demographic characteristics, and related disease history for machine learning model development. AMR, antimicrobial resistance; pwCF, people with cystic fibrosis. *Ciprofloxacin, ceftazidime, meropenem, Pip/Tazo, and tobramycin were defined as the antibiotics that are commonly used in patients with CF. #Sputum cultures in training and validation cohort were from different patients.

Since not all antibiotics are tested for every sputum culture, there will be partially missing results for specific antibiotics in AST. To maximize use of longitudinal information while avoiding the use of future data, we applied within‐patient forward filling (last observation carried forward): when an AST result (resistant/susceptible) was missing for a given culture, we imputed it using the most recent prior AST result for the same antibiotic in the same patient based only on results recorded before that culture date. Cultures with no prior AST result available for that antibiotic (i.e., no value from which to forward fill) were excluded (*n* = 746). Finally, 12,618 sputum cultures and 63,823 days of antibiotic usage in 209 pwCF were included in the analysis (Figure 5).

### 4.4 ML Model Development

The use of various common antibiotics, previous antibiotic resistance histories, lung function during the last hospitalization, demographic characteristics, and medical histories were used to predict AMR. The continuous variables were normalized to the [0,1] interval, while the categorical variables were coded as binary numbers (0 and 1). Sputum cultures were split into training set and testing set at proportions of 8:2 using patient‐level stratified sampling. Specifically, all cultures from the same patient were assigned exclusively to either the training set or the testing set to ensure independence and to prevent data leakage. Stratification was applied to maintain a similar distribution of the outcome (resistant vs. susceptible) across the two datasets. Within the training set, hyperparameter tuning was performed using fivefold cross‐validation constructed at the patient level, ensuring that no patient contributed observations to more than one fold during model optimization. Different ML models were evaluated to optimize predictive performance, consistent with prior work comparing multiple classifiers for antimicrobial susceptibility prediction [33]. To avoid the interference of imputation on the evaluation of model performance, inferred results by forward filling were excluded from the testing set and only used for model training. Sub‐group analyses were performed where cultures were stratified by the presence/absence of *P. aeruginosa*. The contribution of individual predictors was further evaluated by using SHAP analysis.

### 4.5 Statistical Analysis

Clinical characteristics were reported as mean ± standard deviation (SD) for normally distributed variables or median (IQR) for nonparametric distributions and frequencies and proportions for categorical variables. Model performance was evaluated using the area under curve (AUC), precision, recall rate, accuracy, and balanced F score (F1 score). Receiver operating characteristic (ROC) curves were generated using Python 3.7 and Matplotlib 3.0.2. Differences in ROCs between subgroups were compared by Delong test, and two‐sided *p* value < 0.05 was considered statistically significant.

## Author Contributions

Conceptualization: Junrong Jiang, Akhil Naik, and Freddy Frost. Data curation, formal analysis, software, machine learning model development, and visualization: Junrong Jiang and Akhil Naik. Data acquisition, clinical interpretation, and microbiology‐related interpretation: Dilip Nazareth, Dennis Wat, Graham Hyde, Jo Fothergill, and Sarah Benabidallah. Methodology, machine learning supervision, and interpretation of model performance: Sandra Ortega‐Martorell and Ivan Olier. Supervision and critical revision of the manuscript: Gregory Y. H. Lip. Supervision, project administration, and critical revision of the manuscript: Freddy Frost. Writing – original draft: Junrong Jiang and Freddy Frost. Writing – review and editing: All authors. All authors have read and approved the final manuscript.

## Funding

This work was supported by a grant from the UK Health Security Agency (UOL/178192).

## Conflicts of Interest

G. Y. H. L. is a consultant and speaker for BMS/Pfizer, Boehringer Ingelheim, Daiichi‐Sankyo, Anthos. No fees are received personally. He is a National Institute for Health and Care Research (NIHR) Senior Investigator and co‐PI of the AFFIRMO project on multimorbidity in AF (grant agreement No. 899871), TARGET project on digital twins for personalized management of atrial fibrillation and stroke (grant agreement No. 101136244), and ARISTOTELES project on artificial intelligence for management of chronic long‐term conditions (grant agreement No. 101080189), which are all funded by the EU's Horizon Europe Research & Innovation programme. S. O. M. is the PI of the TARGET project and senior investigator in the ARISTOTELES project. I. O. is co‐lead in TARGET and partner lead in ARISTOTELES. F. F. is a speaker for Astrazeneca, Chiesi, and Vertex. F. F. reports speaker fees from Astrazeneca, Chiesi, and Vertex. The remaining authors declare no conflicts of interest.

## Ethics Statement

The study protocol was reviewed and approved by the North‐West Research Ethics Committee and Health Research Authority (approval number: 16/NW/0741). The study was conducted in accordance with the Declaration of Helsinki and UK Health Research Authority guidelines. Written informed consent was waived because this study involved retrospective analysis of fully anonymized, routinely collected clinical data.

## Supporting information




**Figure S1**: SHAP summary plots showing the most important predictors of antibiotic‐specific resistance in P. aeruginosa‐positive cultures.
**Figure S2**: SHAP summary plots showing the most important predictors of antibiotic‐specific resistance in non‐P. aeruginosa cultures.
**Table S1**: The utilization of different antibiotics during the study period for 209 included people living with cystic fibrosis.
**Table S2**: Subgroup analyses in Predicting AMR.
**Table S3**: The frequency of individual antibiotic susceptibility testing and respective resistance among the 13,364 sputum cultures.

## Data Availability

The data that support the findings of this study are available from the corresponding author upon reasonable request. The data are not publicly available due to privacy and ethical restrictions. The individual‐level data analyzed in this study were derived from anonymized routinely collected electronic healthcare records and microbiology surveillance data from a single specialist cystic fibrosis center; therefore, access is subject to institutional governance requirements and appropriate approvals. The analytical code used for data preprocessing, model development, and model evaluation is not publicly available at present; however, further details of the modelling workflow may be available from the corresponding author upon reasonable request.
