# Supplementary material for: Prediction of Antimicrobial Resistance in People Living With Cystic Fibrosis Using Machine Learning
Source: MedComm (2020). 2026 Aug 12;7(8):e70894. doi: 10.1002/mco2.70894 (PMC13462905; doi:10.1002/mco2.70894)
Supplement: Supplementary file 1 [file MCO2-7-e70894-s001.docx]

**Supplementary Materials for**

Prediction of antimicrobial resistance in people living with cystic fibrosis using machine learning

Junrong Jiang^1,2 #^, Akhil Naik^1,5#^, Dilip Nazareth^1,3^, Dennis Wat^1,3^,Graham Hyde^3^, Jo Fothergill^4^, Sarah Benabidallah^1^, Gregory Y. H. Lip^1,5^, Sandra Ortega-Martorell^1,5^, Ivan Olier^1,5^ & Freddy Frost^1,3*^

Corresponding author: Freddy Frost (ffrost@liverpool.ac.uk)

**Institutions:**

1. Liverpool Centre for Cardiovascular Sciences at University of Liverpool, Liverpool John Moores University and Liverpool Heart & Chest Hospital, Liverpool, UK
2. Guangdong Provincial Geriatrics Institute, Guangdong Provincial People’s Hospital (Guangdong Academy of Medical Sciences), Southern Medical University, Guangzhou, China
3. Adult CF Centre, Liverpool Heart & Chest Hospital NHS Foundation Trust, Liverpool, UK
4. Clinical Infection, Microbiology and Immunology, Institute of Infection, Veterinary and Ecological Sciences, University of Liverpool, UK
5. Danish Center for Health ServicesData Science Research, Department of Clinical Medicine, Aalborg Centre, Liverpool John Moores University, Aalborg, Denmark,Liverpool, UK

**The file includes:**

Figure S1 to S2

Table S1 to S3

**Figure S1.** **SHAP summary plots showing the most important predictors of antibiotic-specific resistance in P. aeruginosa-positive cultures.** Panels show feature contributions for predicting resistance to (A) ceftazidime, (B) ciprofloxacin, (C) meropenem, (D) piperacillin/tazobactam and (E) tobramycin.

**Abbreviations:** CAZ, ceftazidime; CIP, ciprofloxacin; CMS, colistimethate sodium; DOT, days of therapy; Freq Diff, frequency difference between resistant and susceptible AST results; LES, Liverpool Epidemic Strain; MDR, multidrug resistance; MEM, meropenem; Res, resistance; SHAP, Shapley Additive Explanations; TOB, tobramycin; TZP, piperacillin/tazobactam; 6m, preceding 6 months; 24m, preceding 24 months; all, the whole available period before the index culture. Other 114 features represents the combined contribution of the remaining features not shown individually**.**

**Figure S2: SHAP summary plots showing the most important predictors of antibiotic-specific resistance in non-P. aeruginosa cultures.** Panels show feature contributions for predicting resistance to (A) ceftazidime, (B) ciprofloxacin, (C) meropenem, (D) piperacillin/tazobactam and (E) tobramycin.

**Abbreviations:** ATM, aztreonam; CAZ, ceftazidime; CIP, ciprofloxacin; CMS, colistimethate sodium; DOT, days of therapy; Freq Diff, frequency difference between resistant and susceptible AST results; MDR, multidrug resistance; MEM, meropenem; Res, resistance; SHAP, Shapley Additive Explanations; TEC, teicoplanin; TOB, tobramycin; TZP, piperacillin/tazobactam; 6m, preceding 6 months; 24m, preceding 24 months; all, the whole available period before the index culture. Other 114 features represents the combined contribution of the remaining features not shown individually.

**Table S1. The utilization of different antibiotics during the study period for 209 included people living with cystic fibrosis**

| **Antibiotic** | **Usage, *median (IQR)*** |
| --- | --- |
| **Colistimethate** |  |
| **Cumulative Usage (days)** | 42 (15– 100) |
| **Annual Usage (d/y)** | 6.3 (3 – 16) |
| **Fosfomycin** |  |
| **Cumulative Usage (days)** | 8 (0 – 35) |
| **Annual Usage (d/y)** | 1.3 (0–8) |
| **Tobramycin** |  |
| **Cumulative Usage (days)** | 25 (7 – 62) |
| **Annual Usage (d/y)** | 4.8 (1 – 10) |
| **Meropenem** |  |
| **Cumulative Usage (days)** | 38 (14 – 99) |
| **Annual Usage (d/y)** | 7.1 (2 – 16) |
| **Ceftazidime** |  |
| **Cumulative Usage (days)** | 24 (6 – 69) |
| **Annual Usage (d/y)** | 4.3 (1 – 12) |
| **Piperacillin/Tazobactam** |  |
| **Cumulative Usage (days)** | 6 (0 – 28) |
| **Annual Usage (d/y)** | 0.9 (0 – 4) |

*Cumulative usage was the sum of days using intravenous antibiotics during the period of follow-up, and the annual usage was calculated by dividing the cumulative usage by the follow-up years. Abbreviations: d/y, days per year; IQR, interquartile range.*

**Table S2. Subgroup analyses in Predicting AMR**

| **AST**  **outcome** | **Observed** | | **Predicted** | | **Prec** | **Rec** | **F1** | **AUC** | **95% CI** |
| --- | --- | --- | --- | --- | --- | --- | --- | --- | --- |
|  |  |  | **S** | **R** |  |  |  |  |  |
| **MDR** | **Pa** | **S** | 784 | 406 | 0.46 | 0.77 | 0.57 | 0.75 | 0.72-0.78 |
|  |  | **R** | 102 | 341 |  |  |  |  |  |
|  | **Non-Pa** | **S** | 458 | 139 | 0.54 | 0.77 | 0.63 | 0.83 | 0.80-0.87 |
|  |  | **R** | 49 | 163 |  |  |  |  |  |
| **CIP** | **Pa** | **S** | 422 | 301 | 0.67 | 0.76 | 0.72 | 0.75 | 0.72-0.79 |
|  |  | **R** | 189 | 615 |  |  |  |  |  |
|  | **Non-Pa** | **S** | 188 | 70 | 0.76 | 0.84 | 0.8 | 0.82 | 0.79-0.86 |
|  |  | **R** | 42 | 224 |  |  |  |  |  |
| **CAZ** | **Pa** | **S** | 582 | 242 | 0.71 | 0.83 | 0.76 | 0.83 | 0.81-0.85 |
|  |  | **R** | 121 | 586 |  |  |  |  |  |
|  | **Non-Pa** | **S** | 7 | 65 | 0.57 | 0.88 | 0.69 | 0.50 | 0.42-0.59 |
|  |  | **R** | 12 | 87 |  |  |  |  |  |
| **MEM** | **Pa** | **S** | 552 | 398 | 0.55 | 0.83 | 0.66 | 0.75 | 0.73-0.78 |
|  |  | **R** | 101 | 481 |  |  |  |  |  |
|  | **Non-Pa** | **S** | 208 | 2 | 0.6 | 0.09 | 0.15 | 0.84 | 0.76-0.93 |
|  |  | **R** | 31 | 3 |  |  |  |  |  |
| **TZP** | **Pa** | **S** | 980 | 282 | 0.40 | 0.71 | 0.51 | 0.80 | 0.76-0.83 |
|  |  | **R** | 77 | 188 |  |  |  |  |  |
|  | **Non-Pa** | **S** | 139 | 10 | 0.68 | 0.55 | 0.61 | 0.83 | 0.75-0.91 |
|  |  | **R** | 17 | 21 |  |  |  |  |  |
| **TOB** | **Pa** | **S** | 559 | 455 | 0.06 | 0.79 | 0.11 | 0.70 | 0.60-0.79 |
|  |  | **R** | 8 | 30 |  |  |  |  |  |
|  | **Non-Pa** | **S** | 1 | 14 | 0.86 | 0.97 | 0.91 | 0.70 | 0.57-0.83 |
|  |  | **R** | 3 | 86 |  |  |  |  |  |

***Abbreviations:*** *AST, antimicrobial susceptibility testing; AUC, area under the receiver operating characteristic curve; CAZ, ceftazidime; CI, confidence interval; CIP, ciprofloxacin; F1, F1 score; MDR, multidrug resistance; MEM, meropenem; Non-Pa, non-P. aeruginosa; Pa, P. aeruginosa-positive; Prec, precision; R, resistant; Rec, recall; S, susceptible; TOB, tobramycin; TZP, piperacillin/tazobactam.*

**Table S3. The frequency of individual antibiotic susceptibility testing and respective resistance among the 13,364 sputum cultures**

| **Antibiotics** | **Tested** | **Resistant** | **Proportion Resistant** |
| --- | --- | --- | --- |
| **Ciprofloxacin** | 10,921 | 6,105 | 55.9% |
| **Meropenem** | 9,290 | 4,175 | 44.9% |
| **Piperacillin/tazobactam** | 8,962 | 2,925 | 32.6% |
| **Ceftazidime** | 8,882 | 5,331 | 60.0% |
| **Tobramycin** | 5,874 | 725 | 12.3% |
| **Gentamicin** | 2,704 | 329 | 12.2% |
| **Fusidic acid** | 2,455 | 238 | 9.7% |
| **Flucloxacillin** | 2,448 | 553 | 22.6% |
| **Erythromycin** | 2,418 | 1,851 | 76.6% |
| **Cefoxitin** | 2,277 | 501 | 22.0% |
| **Penicillin G** | 1,022 | 889 | 87.0% |
| **Trimethoprim** | 472 | 57 | 12.1% |
| **Co-amoxiclav** | 392 | 180 | 45.9% |
| **Ceftriaxone** | 370 | 175 | 47.3% |
| **Colistin** | 294 | 195 | 66.3% |
